# Chronic HSV-1 scalp folliculitis masquerading as scalp dysesthesia in a patient receiving upadacitinib

**DOI:** 10.1016/j.jdcr.2026.09.024

**Published:** 2026-09-22

**Authors:** Karen Koch, Duncan Lamont

**Affiliations:** a Dermatology Department, Te Whatu Ora Health Waikato (Waikato Hospital), Hamilton, New Zealand; b Honorary Senior Clinical Lecturer, Waikato School of Medicine (Auckland University), Hamilton, New Zealand; c Pathology Department, Te Whatu Ora Health Waikato (Waikato Hospital), Hamilton, New Zealand

**Keywords:** atopic dermatitis, herpes simplex virus 1, herpetic folliculitis, immunosuppression, Janus kinase inhibitor, scalp, upadacitinib

## Introduction

HSV-1 folliculitis of the scalp is rarely reported and can be clinically misleading when vesicles or pustules are absent, as they often are in follicular infections.^1^, ^2^, ^3^ Diagnosis is frequently made only after antibacterial, antifungal, and anti-inflammatory treatments have failed,^2^^,^^4^^,^^5^ and histopathology may be nonspecific: folliculocentric lymphohistiocytic inflammation and keratinocyte apoptosis are the dominant findings, with classic viral cytopathic changes often absent.^3^^,^^6^, ^7^, ^8^ We report a case in which all of these factors converged, with the added complexity of the introduction of Janus kinase (JAK) inhibitor therapy, resulting in a 4-month diagnostic delay.

## Case report

A 63-year-old man had a past medical history of severe atopic dermatitis and seborrhoeic eczema treated with long-term methotrexate and ciclosporin, multiple non-melanoma skin cancers, and previous herpes simplex infection at a non-scalp site. He was referred to the dermatology clinic with persistent scalp itch of approximately 8 months’ duration that had not responded to topical corticosteroid scalp preparations or ketoconazole shampoo, prescribed after a scraping grew Malassezia. On examination, there was an enlarging, firm, annular, scaly plaque on the occipital scalp measuring approximately 6 × 6 cm with a raised edge (Fig 1).

**Fig 1:**
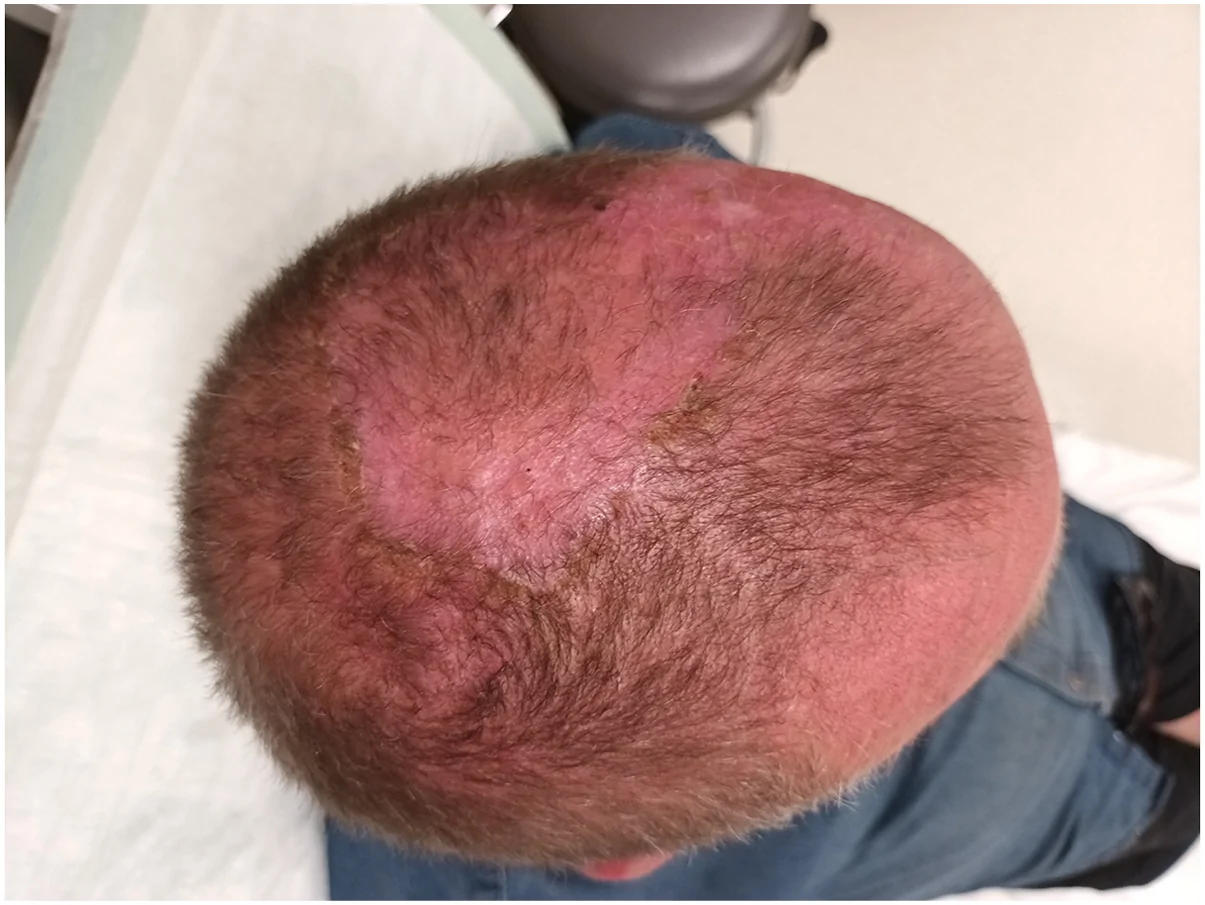
Annular scaly plaque on the occipital scalp at initial dermatology consultation.

Punch biopsy from the left occipital scalp showed sun-damaged skin with mild hyperkeratosis. The dominant abnormality was a deep perifollicular lymphohistiocytic infiltrate with a sparse neutrophilic component (Fig 2 *A*; H&E, original magnification ×100). Affected pilosebaceous units showed striking epithelial apoptosis involving outer root sheath keratinocytes (Fig 2, *B*; H&E, ×200). Periodic acid–Schiff staining demonstrated subtle filamentous structures within a hair shaft, initially interpreted as possible branching bacteria (Fig 2, *C*; PAS, ×200). There was no dysplasia or malignancy. Herpetic infection had not been included in the clinical differential diagnosis and no lesional swab had been taken at that stage; the possibility was first raised by the reporting pathologist on the basis of the follicular epithelial apoptosis. HSV PCR performed on the formalin-fixed, paraffin-embedded biopsy specimen was negative, and was interpreted at the time as excluding herpetic infection.

**Fig 2:**
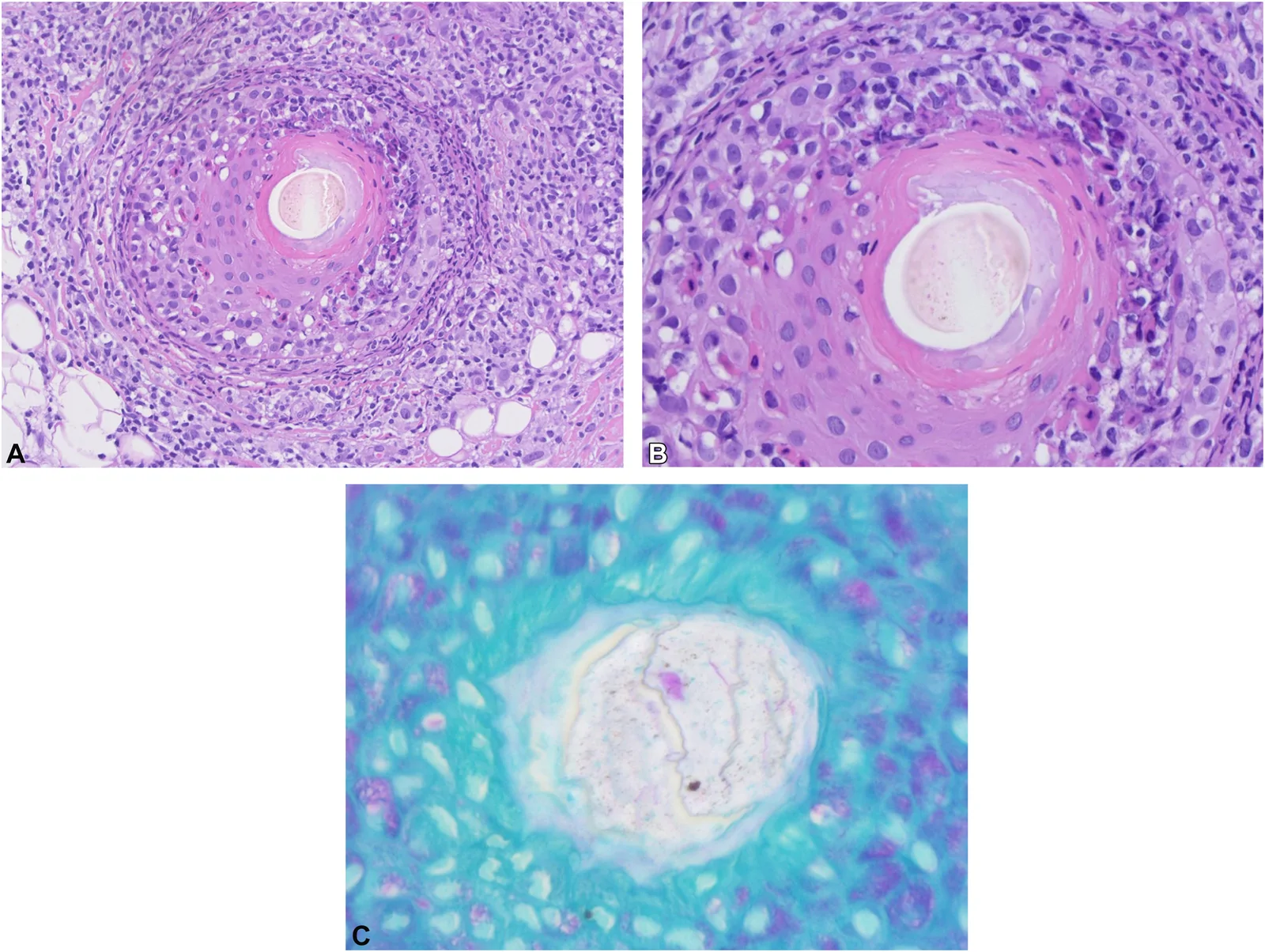
Histopathological findings of the scalp punch biopsy. **A,** Deep perifollicular lymphohistiocytic infiltrate surrounding a pilosebaceous unit; hematoxylin and eosin (H&E), original magnification ×100. **B,** Striking outer root sheath epithelial apoptosis involving the pilosebaceous unit; note the absence of classic viral cytopathic changes such as ballooning degeneration or multinucleation; H&E, ×200. **C,** Periodic acid–Schiff (PAS) stain demonstrating subtle filamentous material within a hair shaft, initially interpreted as possible branching bacteria; PAS, ×200.

**Fig 3:**
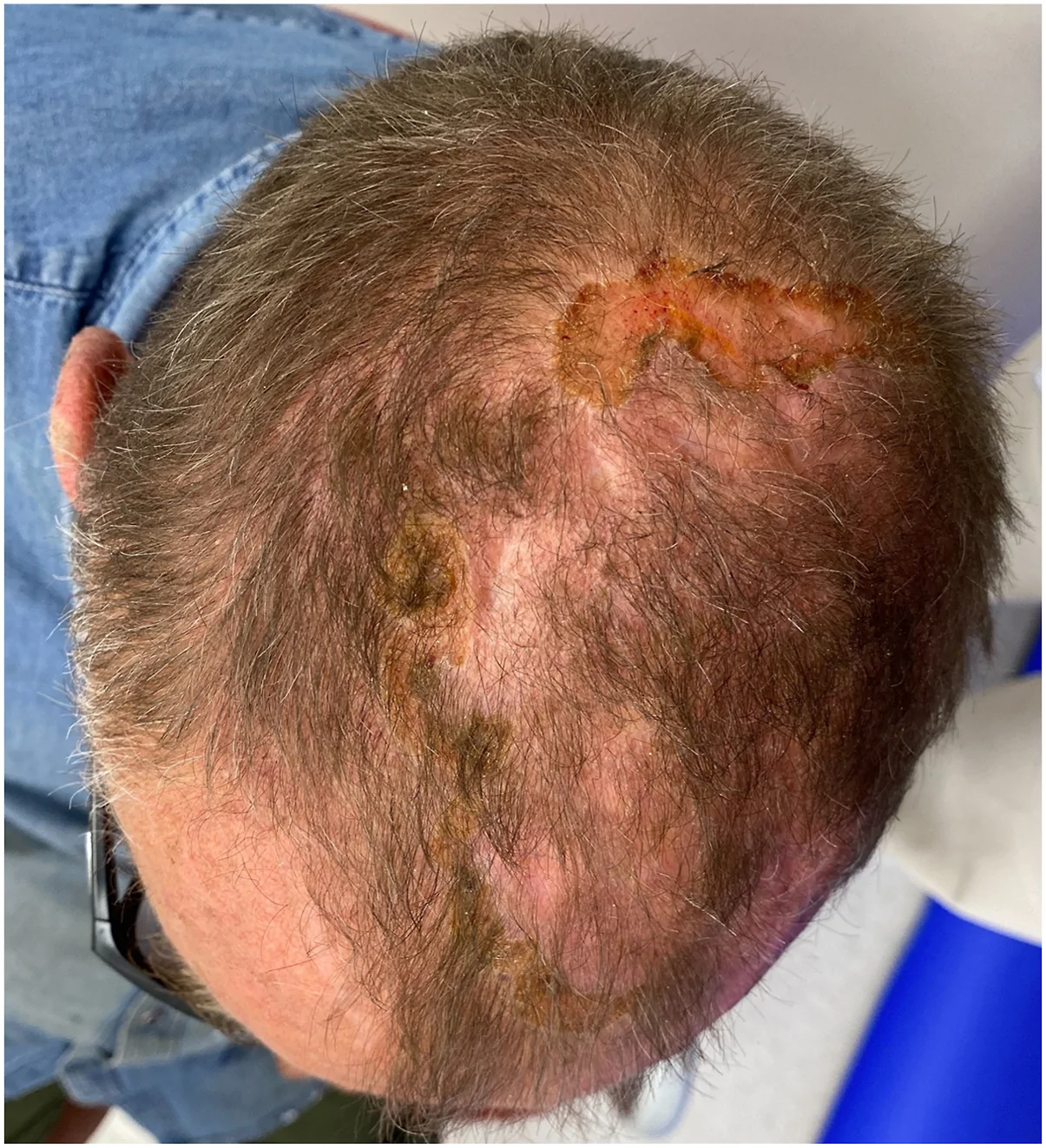
Worsening erosions within 3 months of starting upadacitinib.

**Fig 4:**
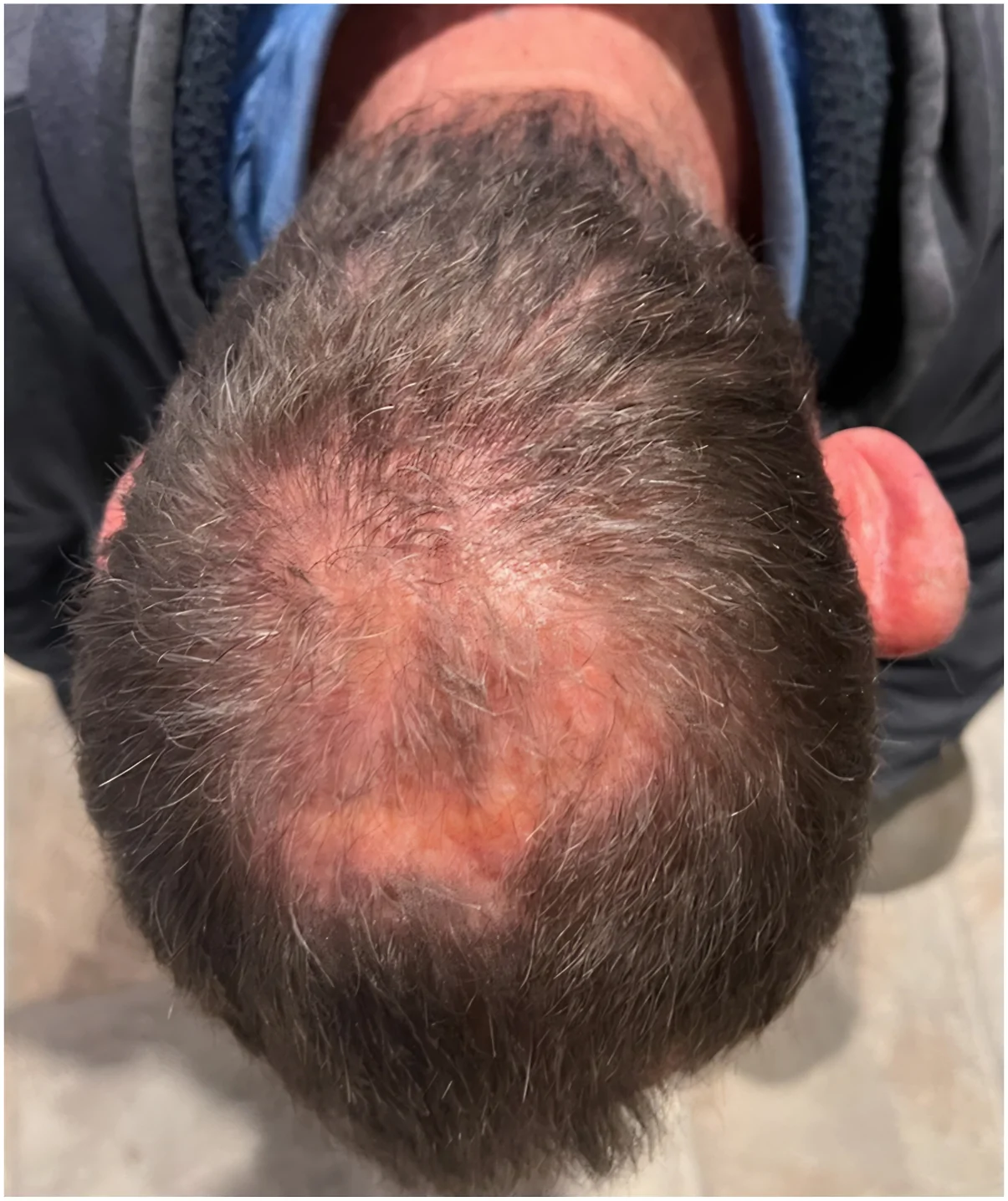
Healing of the scalp within 6 weeks of initiating valaciclovir 1 g three times daily.

Bacterial tissue culture grew Staphylococcus aureus, whereas Nocardia culture and mycobacterial studies were negative. Fungal investigations were discordant: microscopy was negative, but cultures later isolated Didymella species and Lichtheimia corymbifera, organisms considered of uncertain significance or possible contaminants. The patient was sequentially treated with oral antibiotics, oral itraconazole, topical antifungal therapy, topical corticosteroids, chlorhexidine wash, mupirocin, and, subsequently, low-dose isotretinoin, without any change in symptoms.

Systemic therapy for atopic dermatitis was changed to upadacitinib 1 month after the initial dermatology consultation, with excellent improvement in eczema. However, the scalp lesion persisted. At 3 months, the annular plaque had evolved into a flat, non-healing circular erosion with severe burning and itching, rated at 8/10 (Fig 3). Cervical spine disease and neuropathic scalp dysesthesia were considered, and symptomatic treatments included pregabalin, topical anesthetic, and intralesional triamcinolone. Persistent erosions, now present for approximately 4 months after the initial consultation, prompted a swab of the base of the erosion for HSV and VZV PCR.

The swab was positive for HSV-1 DNA by PCR, and the patient was treated with valaciclovir 1 g three times daily for 2 weeks, with rapid symptom resolution. The lesion healed over 6 weeks, leaving residual scarring alopecia (Fig 4).

## Discussion

Several features of this case obscured the diagnosis: an annular, scaly plaque without vesicles or pustules; the chronicity of the lesions; a biopsy demonstrating only perifollicular lymphohistiocytic inflammation with follicular keratinocyte apoptosis; cultures yielding organisms of uncertain significance; and a negative HSV PCR on tissue. Each finding argued against herpetic infection, and together they contributed to a 4-month diagnostic delay during which the plaque eroded and the pain became severe enough to prompt consideration of neuropathic scalp dysesthesia.

Nonspecific histopathology is characteristic of herpetic folliculitis, which does not typically produce the grouped vesicles or short-lived ulcers of cutaneous herpes simplex at other sites. In a series of 21 PCR-confirmed cases, Boër et al identified ballooning degeneration and multinucleated giant cells in fewer than half, and steel-grey nuclei in only 6; lymphocytic folliculitis, perifolliculitis, and necrotic follicular keratinocytes were the most consistent findings.^3^ Crowson et al reported comparable findings in 28 patients, predominantly with varicella zoster virus, in whom dense folliculocentric lymphohistiocytic infiltrates predominated and classic cytopathic change was the exception.^6^ In retrospect, the outer root sheath apoptosis in our specimen was consistent with herpetic folliculitis, but with the negative tissue PCR it was not considered diagnostic.

A negative tissue PCR does not exclude herpes and was overlooked. Herpetic folliculitis is a focal process centered on the pilosebaceous unit, and the punch biopsy was taken from the scaly margin of an intact plaque rather than from actively eroded skin. We likely sampled an area of minimally involved follicle. Sensitivity is further reduced by formalin fixation and paraffin embedding, which fragment and cross-link nucleic acid and introduce PCR inhibitors, and negative tissue PCR has previously been reported in histologically suspicious herpetic folliculitis.^3^ The later swab sampled the base of an active erosion, where viral shedding is greatest. For suspected cutaneous herpetic infection, PCR performed on a swab of the base of an unroofed vesicle, erosion, or ulcer should always be the first-line diagnostic test. It is inexpensive, non-invasive, rapidly reported, and more sensitive than PCR on formalin-fixed tissue. Biopsy retains an important role in excluding alternative diagnoses, but a negative tissue PCR should not be taken to exclude herpetic infection when clinical or histological suspicion persists.

The microbiological isolates contributed further to the delay. Neither the Staphylococcus aureus nor the fungal isolates were supported by the histological findings, and neither predicted the response to treatment; both are most likely to have represented colonization or contamination. Earlier viral swabbing, at the point of the first treatment failure, would have shortened the diagnostic interval considerably.

The patient was already substantially immunosuppressed before upadacitinib was introduced, with longstanding atopic dermatitis, several years of methotrexate and ciclosporin, and topical corticosteroid use on the scalp. The addition of a selective JAK1 inhibitor is nonetheless likely to have been relevant. In the phase 3 AD Up trial, oral herpes occurred in 3% of patients receiving upadacitinib 15 mg and 8% of those receiving 30 mg, herpes zoster occurred in 1% to 2% of treated patients over 16 weeks, and eczema herpeticum was reported in the upadacitinib arms but not with placebo.^9^ JAK1 inhibition impairs interferon signaling and T-cell antiviral responses; in an already immunocompromised patient this may permit herpetic infection to persist in an attenuated, non-vesicular form rather than presenting acutely.

The burning pain and the erosive, non-healing character of the lesion were initially attributed to repeated scratching and picking, and the severity of the neuropathic-type pain prompted cervical spine investigation, pregabalin, and intralesional triamcinolone before herpetic infection was reconsidered. The only previously reported case of HSV-1 scalp folliculitis presented as crusted papules, without erosion or pain.^1^ The dysesthetic phenotype observed here may reflect deeper follicular and perineural involvement in the setting of sustained immunosuppression.

In refractory scalp folliculitis, particularly when the lesion is painful, burning, erosive, crusted, annular, or non-healing, HSV and VZV PCR on a lesional swab should be requested as part of the initial investigation. Histopathology may not demonstrate the classic features of herpetic infection, immunosuppressive therapy may modify the clinical presentation, and a negative tissue PCR does not exclude the diagnosis.

## Conflicts of interest

None disclosed.

## References

[1] Collins J., Hahn P. (2025). A rare case of herpes simplex virus 1 folliculitis on the scalp of a military member. JAAD Case Rep.

[2] Jang K.A., Kim S.H., Choi J.H., Sung K.J., Moon K.C., Koh J.K. (2000). Viral folliculitis on the face. Br J Dermatol.

[3] Boër A., Herder N., Winter K., Falk T. (2006). Herpes folliculitis: clinical, histopathological, and molecular pathologic observations. Br J Dermatol.

[4] Foti C., Calvario A., d'Ovidio R. (2005). Recalcitrant scalp folliculitis: a possible role of herpes simplex virus type 2. New Microbiol.

[5] Cao Z., Zhang C., Wei J., Wei G. (2022). Facial herpetic folliculitis should be concerned in the clinic: a retrospective case series. Clin Cosmet Investig Dermatol.

[6] Crowson A.N., Saab J., Magro C.M. (2017). Folliculocentric herpes: a clinicopathological study of 28 patients. Am J Dermatopathol.

[7] Nakagawa K., Kishida M., Kimura Y. (2014). Plaque type herpetic folliculitis involving the eccrine gland: immunohistochemical analysis. J Dermatol.

[8] Daruish M., Cazzato G., Markiewicz D., Taibjee S., Fortarezza F., Calonje E. (2024). A review of atypical cutaneous histological manifestations of herpes zoster. Viruses.

[9] Reich K., Teixeira H.D., de Bruin-Weller M. (2021). Safety and efficacy of upadacitinib in combination with topical corticosteroids in adolescents and adults with moderate-to-severe atopic dermatitis (AD Up): results from a randomised, double-blind, placebo-controlled, phase 3 trial. Lancet.

